# Cost-Effectiveness Analysis from a Randomized Controlled Trial of Tailored Exercise Prescription for Women with Breast Cancer with 8-Year Follow-Up

**DOI:** 10.3390/ijerph17228608

**Published:** 2020-11-19

**Authors:** Louisa G. Gordon, Elizabeth G. Eakin, Rosalind R. Spence, Christopher Pyke, John Bashford, Christobel Saunders, Sandra C. Hayes

**Affiliations:** 1 Population Health Department, QIMR Berghofer Medical Research Institute, Locked Bag 2000, Royal Brisbane Hospital, Brisbane, Qld 4029, Australia; 2 School of Nursing, Queensland University of Technology (QUT), Kelvin Grove, Brisbane, Qld 4059, Australia; 3 Faculty of Medicine, School of Public Health, The University of Queensland, Herston, Brisbane, Qld 4006, Australia; e.eakin@uq.edu.au; 4 Menzies Health Institute Qld, Griffith University, Nathan, Brisbane, Qld 4111, Australia; r.spence@griffith.edu.au (R.R.S.); sandi.hayes@griffith.edu.au (S.C.H.); 5 Mater Public and Private Hospital, South Brisbane, Qld 4101, Australia; Christopher.Pyke@mater.org.au; 6 The Wesley Hospital, Auchenflower, Brisbane, Qld 4066, Australia; j.bashford@mac.com; 7 Faculty of Health and Medical Sciences, University of Western Australia, Perth, WA 6009, Australia; christobel.saunders@uwa.edu.au

**Keywords:** cost-utility analysis, cost-effectiveness analysis, exercise, breast cancer

## Abstract

Studies show conflicting results on whether exercise interventions to improve outcomes for women with breast cancer are cost-effective. We modelled the long-term cost-effectiveness of the *Exercise for Health* intervention compared with usual care. A lifetime Markov cohort model for women with early breast cancer was constructed taking a societal perspective. Data were obtained from trial, epidemiological, quality of life, and healthcare cost reports. Outcomes were calculated from 5000 Monte Carlo simulations, and one-way and probabilistic sensitivity analyses. Over the cohort’s remaining life, the incremental cost for the exercise versus usual care groups were $7409 and quality-adjusted life years (QALYs) gained were 0.35 resulting in an incremental cost per QALY ratio of AU$21,247 (95% Uncertainty Interval (UI): Dominant, AU$31,398). The likelihood that the exercise intervention was cost-effective at acceptable levels was 93.0%. The incremental cost per life year gained was AU$8894 (95% UI Dominant, AU$11,769) with a 99.4% probability of being cost effective. Findings were most sensitive to the probability of recurrence in the exercise and usual care groups, followed by the costs of out-of-pocket expenses and the model starting age. This exercise intervention for women after early-stage breast cancer is cost-effective and would be a sound investment of healthcare resources.

## 1. Introduction

Breast cancer is the most common cancer among women worldwide. By 2040, an estimated 3.1 million people will be diagnosed with breast cancer, an increase of 47% from 2018 [1]. The annual number of deaths worldwide are expected to rise to 991,904 by 2040 [1]. Although 5-year survival rates for localized breast cancer are high at ~95% [2], incidence is also high (one in seven women will develop breast cancer [3]) with key lifestyle risk factors remaining widespread and growing. Risk factors for postmenopausal breast cancer include obesity, having fewer children, low levels of physical activity, reduced levels of breastfeeding, and having children later in life [4].

Survivors of breast cancer and its treatment can experience reduced quality of life that lingers well after diagnosis and treatment. Long-term effects can include fatigue, pain, cognitive limitations, menopausal symptoms, depression, fear of recurrence, sleeping problems, and decreased physical fitness [5]. Increased fatigue and decreased physical fitness can lead to longer absences from work and delayed return to everyday activities [6]. Hence, the need to improve the quality of life of breast cancer survivors is especially important as their numbers increase.

Physical activity is advocated by cancer agencies and public health organizations to reduce the impacts of symptoms and side-effects from cancer and its treatment [7,8,9,10,11]. Evidence supports the beneficial effects of physical activity during breast cancer treatment and beyond, particularly when the exercise dose exceeds 150 min of moderate intensity, mixed mode exercise per week [10]. Moreover, by alleviating side-effects and improving recovery, systematic reviews consistently show that physical activity reduces breast cancer mortality and events, and all-cause mortality [12,13,14,15]. In 2011, a randomized controlled trial of a tailored exercise intervention, *Exercise for Health* (EfH)*,* was undertaken in Brisbane, Australia for newly diagnosed women with early-stage breast cancer [16,17]. At 18 months, the intervention group had superior quality of life and fitness and lower fatigue than women in the control group [17]. At 8-years follow-up, significant increases in overall and breast-cancer survival were observed in the intervention group, adjusted for cancer stage [18].

Despite the mounting evidence on the health benefits of exercise interventions for breast cancer, relatively little is known about their economic value to inform decisions about wider uptake into routine care [19]. Six economic evaluations have assessed the costs and effects of physical activity interventions for women with breast cancer [20,21,22,23,24,25]. While most were based on randomized controlled trials, the majority modelled short-term economic benefits and the findings were contradictory. The purpose of this study is to undertake a cost-effectiveness analysis using long-term 8-year follow-up data and model the expected longer-term consequences of the EfH intervention. 

## 2. Materials and Methods 

### 2.1. Overview

We undertook a Markov cohort model for the cost-effectiveness analysis and modelled women with early stage breast cancer over their remaining lifetime. The measure of benefit used was quality-adjusted life years (QALYs), a generic metric that combines survival with quality of life, commonly used for economic evaluations. Costs and QALYs were aggregated in yearly cycles and compared across the exercise intervention and usual care strategies. Data inputs were obtained from the EfH-randomized controlled trial, supplemented with epidemiological, quality of life, and healthcare cost studies. We adhered to the Consolidated Health Economic Evaluation Reporting Standards (CHEERS) statement for reporting economic evaluations and good practice guidelines for decision-analytic modelling for healthcare interventions [26,27].

### 2.2. Intervention and Comparator 

The exercise intervention was based on the EfH trial conducted between October 2006 and June 2008 in Brisbane, Australia. EfH was a randomized controlled trial evaluating an 8-month exercise program for women after surgery for primary breast cancer. Women were recruited from four hospitals (*n* = 194). On average women were aged 52.4 years (standard deviation 8.5), were overweight (mean body mass index 26.6 ± 5.2 kg/m^2^), 92.3% had Stage I or II disease [28]. Personal and diagnostic characteristics were similar across the groups [28]. Full details are available [28] but briefly, the intervention involved 16 sessions with an exercise physiologist across the 8-month intervention. The aim of the sessions is to support women to be exercising at least four days per week for 45 min, including aerobic and resistance-based exercise, with individual prescriptions tailored to each woman’s ability. The comparator or “usual care” group received no specific intervention but may have received information related to exercise following breast cancer from health professionals or other resources during the course of their health care [28]. This may have included undertaking exercise on the women’s own accord, seeking private professional exercise services or not doing any exercise by preference. All participants gave their informed consent for inclusion before they participated in the study. The study was conducted in accordance with the Declaration of Helsinki, and the EfH trial (ACTRN: 012606000233527) was approved by the Human Research Ethics Committee at the Queensland University of Technology and each of the four participating hospitals. 

### 2.3. Model Structure

**Figure 1:**
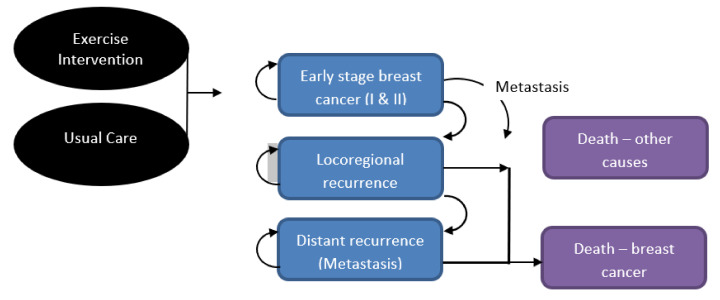
Illustration of Markov model health states.

A Markov cohort model was designed with five mutually exclusive health states (Figure 1) starting in the “early stage I and II breast cancer” health state. Women either remained there or, following disease progression, moved to locoregional or metastatic cancer health states and/or death from breast cancer. At any time, the women could die of other causes. Movement between health states occurred by transition probabilities. Relevant costs and health utilities (similar to quality of life) were assigned to each health state and aggregated over time. 

### 2.4. Data Inputs

Full details of model inputs are summarized in Table 1 and calculations are provided in the Supplementary file.

Intervention-related variables: Model variables relating to the EfH intervention arm (Table 1 for details) included the additional intervention costs, the probability of all-cause mortality and breast cancer mortality for women in the exercise intervention, recurrence rates, and the health utility gain attributed to the exercise intervention. Parallel variables for the usual care arm were applied. All other variables in the model applied commonly to both comparison arms.

Transition probabilities: The model transition probabilities between the health states and events used data from the EfH trial and published literature of large, long-term, and recent studies (Table 1). Formulas were used to convert rates into one-year probabilities [42]. The age-specific probabilities of death from all causes was derived from the female age-specific mortality rates recorded by the Australian Bureau of Statistics [32]. Locoregional and distant recurrence rates were obtained from a retrospective study conducted by Wu et al. (2016) with a median follow-up of 6.9 years [29]. The probability of dying from breast cancer was calculated using the 10-year survival rate from the study conducted by Witteveen et al. (2015) [34] (women were diagnosed three years earlier than women in the EfH study) and US Surveillance, Epidemiology, and End Results data [33]. The probability of developing metastatic cancer after locoregional disease was a weighted average from node negative and node positive cohorts [30,31].

Health utilities: Utilities are used to calculate QALYs, by applying utility weights (scored from 0 =worst health to 1 = full health) to the length of life remaining. Health utilities were directly obtained from the EfH trial with improvements reported for both the intervention (0.07) and usual care groups (0.02) over the trial period [20]. These were applied only for the duration of the trial and no assumption of ongoing benefit was made. Utilities for women with breast cancer at pre-diagnosis and at locoregional, metastatic, and terminal disease were extracted from a systematic review of health utilities in breast cancer by Paracha et al. (2016) [41] (Table 1). The pre-diagnosis utility was assigned to the early stage breast cancer health state and the others applied accordingly.

Costs: A societal cost perspective was employed considering healthcare system, patient, and carer economic burdens. Intervention costs are previously detailed in [20] and included professional labour, administration, educational materials, office rental, phone charges for program delivery, equipment, and travel costs (Supplementary File). The costs of breast cancer follow-up care, costs of treatment for recurrence, and productivity losses were included. Follow-up care costs were calculated from resource use expected using recommendations for follow-up of women with early breast cancer [36]. General practitioner visits, specialist visits, and mammograms were valued using Medicare Benefit Scheme item pricing. The costs of treating a recurrence were derived from Verry et al. (2012) [35]. Women who experienced a recurrence were assumed to complete treatment in a single one-year cycle. Healthcare costs for end-of-life care differed depending on if women died of breast cancer or other diseases reported in an Australian study by Reeves et al. (2017) [37]. The intervention was not expected to influence primary care and adjuvant treatments and these costs were not included.

Out-of-pocket costs were estimated for follow-up treatment and lost income for patients and their families while recovering from breast cancer or experiencing a recurrence. Productivity losses were estimated by hours of time off work from a recent Australian report [38] and adjusted for female labour force participation rates and age. Average Australian salaries for women were applied to estimate the costs [40] and the proportion of women of working age who work incorporating those who work part- and full-time hours. Productivity lost was also included for women who died prematurely from breast cancer, up to the retirement age of 65 years, based on Australian estimates by Carter et al. (2016) [39].

### 2.5. Analyses 

The main outcomes were mean costs, QALYs and life-years calculated from 5000 Monte Carlo simulations. An incremental cost-effectiveness ratio (ICER) was calculated as the difference in costs of the two strategies divided by the difference in QALYs or life years. An annual discount rate of 5% was applied to costs, QALYs and life-years as recommended by the Australian Government for outcomes extending beyond one year [43]. A half-cycle correction was applied to outcomes in the initial and final cycles as transitions could have occurred in the middle of each cycle. Costs were adjusted for inflation where necessary to 2019/2020 Australian dollars.

One-way sensitivity analyses were performed to calculate the ICERs using plausible variation in input values, re-running simulations 5000 times for each variable change. The 95% confidence intervals (CIs) of input values were used where available or ±10% variation in mean values. Probabilistic sensitivity analyses were undertaken to assess the likelihood of the exercise intervention being cost-effective, considered at a willingness-to-pay threshold of AU$50,000 per QALY gain. Input variables were assigned beta, gamma, and log normal distributions where appropriate (Supplementary File). To obtain the 95% uncertainty interval (UI), we used the percentile method, ranking the incremental costs, QALYs, and ICERs and removing the top and bottom 2.5%. Analyses were performed in TreeAge Pro Healthcare 2020 R2.1 software.

## 3. Results

**Figure 2:**
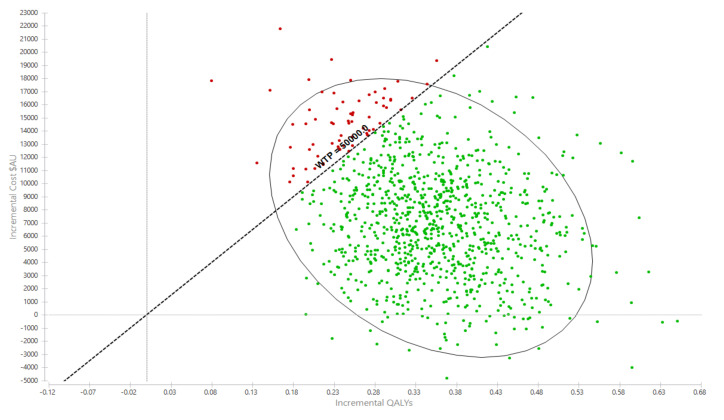
Probabilistic sensitivity analyses, incremental cost per QALY gain scatterplot. AU$ = Australian dollars, QALY = quality-adjusted life year.

**Figure 3:**
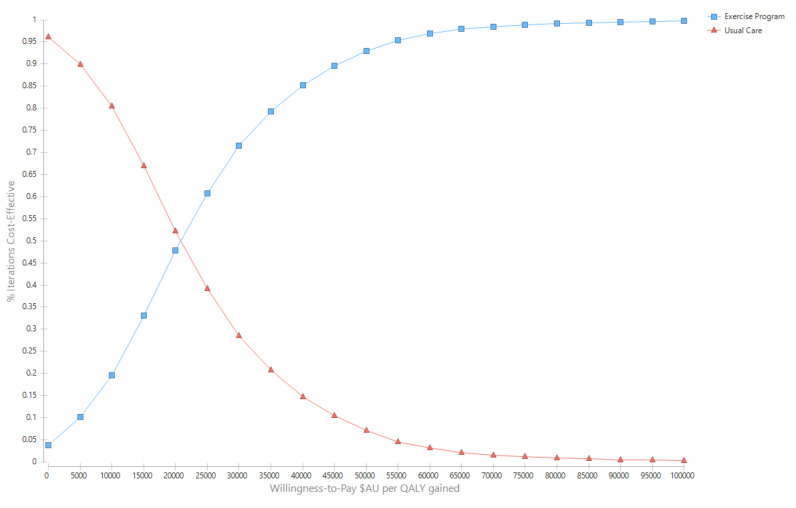
Cost-effectiveness acceptability curves.

Over the cohort’s remaining life, the mean cost for the exercise group was AU$281,445 (95% UI $137,890, $372,701) compared with AU$274,035 (95% UI $135,309, $362,044) for the usual care group (Table 2). The corresponding QALYs were 10.97 (95%UI 8.56, 12.93) for the exercise group and 10.63 (95%UI 8.26, 12.51) for usual care. The incremental cost per QALY gained for the exercise intervention was AU$21,247 (95%UI Dominant, $31,398) (Table 2). The likelihood that the exercise intervention was cost-effective was 93.0% (Figure 2 and Figure 3).

Interpretation: Each dot represents an incremental cost and incremental life year pairing using the assigned distributions around each model parameter, selected randomly during 5000 iterations. Dots falling to the right of the diagonal line (the willingness-to-pay threshold of AU$50,000 per quality-adjusted life year gained) are cost-effective. The proportion of simulations cost-effective is 93.0%. The oval is the 95% ellipse and represents the 95% uncertainty interval.

Results for the outcome ‘life-years saved’ were more cost-effective than using QALYs as the outcome. The incremental cost per life year saved was $8894 (95% UI Dominant, $11,769) with a 99.4% probability of being cost effective.

**Figure 4:**
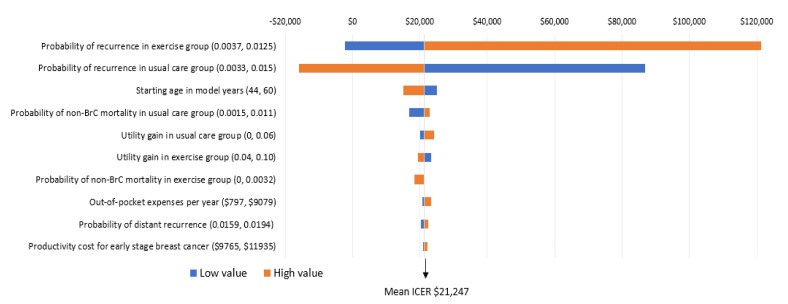
One-way sensitivity analyses; incremental cost per QALY gain.

The key factors influencing the results are the risk of breast cancer recurrence, patient out-of-pocket costs, and patient age. Modifications to risk of recurrence in the exercise and usual care groups was the only factor that influenced the incremental cost per QALY ratio in a meaningful way (Figure 4). Specifically, when the probability of recurrence in the exercise group was high (0.0125), the potential cost-effectiveness of the intervention is less (that is, would no longer be considered cost-effective). In contrast, when probability of recurrence is higher than what was defined in the foundation model (which is likely), the intervention becomes particularly cost-effective (that is, would reduce the incremental cost per QALY ratio by $23,596 over the long term) (Figure 4). While modification to other factors including age at diagnosis, non-breast cancer mortality, and health-related quality of life also influenced findings (making exercise more or less cost-effective), the incremental cost-effectiveness ratios remained below the cost-effective threshold of $50,000 (Figure 4).

Interpretation: The importance of each variable on the ICER (incremental cost per QALY ratio) is presented from top to bottom. The high and low values for each variable are presented in brackets. The tails of each bar represent the maximum and minimum ICER for each variable. The vertical line indicates the ICER from the base case ($21,247) to provide a reference for the changes. Each high and low value was altered and 5000 simulations re-run to obtain these ICERs.

## 4. Discussion

Our analyses show that a tailored exercise intervention for women during and beyond treatment for early stage breast cancer is likely to be cost-effective in the Australian healthcare setting. That is, the benefits associated with exercise intervention were sufficient to warrant their additional costs. Our analysis included wider social costs accounting for time off work by patients and/or their carers and out-of-pocket costs for families, which reflect a broad view of the true costs of breast cancer. We took a conservative view for benefits by assessing QALYs and life years saved and made no assumptions about sustained quality of life or longevity after the 8-year follow-up period ended.

The benefits for women in the exercise group may be explained through improved health-related quality of life, improved progression-free survival or from higher overall survival, which the EfH trial suggests are robust long-term outcomes of exercise. Evidence shows that exercise programs are beneficial across all these effects [8,13]. The 8-year follow-up data from the EfH trial demonstrated that mixed-type, moderate-intensity exercise was favorable to disease recurrence and overall survival outcomes, irrespective of age, stage of disease, presence of other comorbidities, or body mass index [18]. Although the EfH trial was not powered or designed to detect significant group differences in these survival outcomes, evidence from meta-analyses also support these findings (overall survival hazard ratio 0.59, 95%CI: 0.53–0.65, *p* < 0.01 across 6 cohort studies) [13,44]. Overall survival is protected by regular exercise through improved physiological functioning in the immune, hormone, musculoskeletal, and cardiovascular systems and through protective psychological effects in reducing anxiety and depression [12,13,14,15]. For women who have had chemotherapy or adjuvant radiotherapy, exercise can counteract the late effects of cardiotoxicity caused by damage to the chest and heart [45]. In addition, since most women in the study had at least one or more comorbidities at baseline, it is plausible that women who were exercising in the EfH trial had improvements in cardiovascular disease, osteoporosis, and Type 2 diabetes.

Six cost-effectiveness studies have been reported on exercise programs for women following breast cancer diagnosis [20,21,22,23,24,25]. It is difficult to make clear comparisons across the studies due to their different health systems, and for interventions with different components, durations, and exercise intensities. Nonetheless, these studies show mixed results but all showed reasonably low costs for the exercise interventions with one having cost savings [24]. In general, overall healthcare costs between the intervention and control groups were similar, meaning in most cases, the intervention paid for itself by creating healthcare cost-offsets. Although three studies found favourable cost-effective findings [23,24,25], those with more questionable economic efficiency showed insufficient benefit for exercise rather than being costly. There are several possible explanations for exercise having relatively small benefits in cost-effectiveness studies. Despite the wide support and recommendation by decision-making bodies to use QALYs in cost-effectiveness analysis, this metric may be insensitive when marked survival differences are not expected, or when symptom severity or change in severity of symptoms are unlikely to be reasonably captured using the EuroQoL-5D health utility tool. In this particular population, women have early stage breast cancer with good survival prospects and similar quality of life post-breast cancer (compared with age-matched norms) making it more difficult to demonstrate QALY gains. Furthermore, the potential for contamination in control groups, as was observed in two studies [20,22], also influences cost-effectiveness findings in the conservative direction (that is, makes it less likely to find an exercise intervention cost-effective). Of note, it is also known in the wider exercise oncology base, that participants who have been physically active pre-cancer are more likely to participate in exercise trials and that therefore participants randomized to the control group of an exercise oncology trial are more likely to engage in exercise of their own accord than those who do not participate in exercise trials [46]. In another study, the intervention consisted of a low dose intervention (i.e., 3 contacts over 12 months) which is likely to be insufficient to show effectiveness [21]. Taken together with their substantial heterogeneity, and conservative approaches to valuation, these studies suggest that exercise interventions are beneficial across various outcome measures and low cost to society. These findings potentially highlight the inadequacy of the current public reimbursement system in Australia, which caps the number of sessions with an allied health professional over 12 months at five, with these sessions needing to be shared among all allied health professionals.

In broad terms, physical inactivity incurs a massive economic cost in Australia, estimated up to $850 million in healthcare expenditure and $15.6 billion in lost production, per year [47]. Despite this, public health investment in disease prevention through promotion of healthy behaviors is challenging when upfront outlays are needed and expected health benefits are delayed. However, prevention is an essential component of an effective health system. Compared with other developed countries whose governments spend 2–4% of health expenditure on prevention, Australia has below-average prevention investment (1.5%) [48]. Worryingly for many countries, a large proportion of spending in prevention is spent on less cost-effective measures [48] and economic evaluations are required to inform decision-makers of the “best buys.” For example, evidence on national breast cancer screening generally confirms its cost-effectiveness, and would be further improved with risk-based approaches that also reduce over-diagnosis, but there is less consensus on the cost-effectiveness of general health check-ups [48].

The strengths of this work include using high-quality randomized controlled trial evidence (with intention-to-treat analysis, complete follow-up data), robust data from large recent studies and, the model’s lifetime analytical horizon. This model has likely underestimated the benefits to women’s health from the exercise intervention, particularly relating to reductions in poor health effects from comorbid conditions. In addition, potential benefits from exercise may have arisen from the reduced need for primary care, reduced risk of dose reductions and delays associated with adjuvant treatment, and reduced risk of new breast cancer. Therefore, we consider our analysis to be conservative. A societal viewpoint of costs were taken to capture the full implications of productivity losses and family disruptions. Limitations of this study include the reliance on international data estimates when Australian epidemiological statistics were not available and the use of breast cancer recurrence estimates (based on small numbers) from the EfH trial. Whether the impact of chemotherapy completion has positive longer-term benefits also requires more evidence to confirm this definitively and this model could be updated in due course.

As with many health promotion programs, often the most disadvantaged groups are more vulnerable to poorer health outcomes while also being the least likely to participate. One element of socioeconomic disadvantage is living in rural or remote locations with less access to healthcare services. The evaluated exercise program was delivered remotely to women residing rurally via telehealth, which had the dual benefits of reaching a broad population geographically while keeping patient out-of-pocket costs and time off work low. Further research could assess whether telehealth platforms are feasible for follow-up maintenance programs and for delivering care to other cancer populations.

## 5. Conclusions

The EfH tailored exercise intervention for women after breast cancer diagnosis is cost-effective compared with usual care. Investing in resources to facilitate prescribed regular exercise in this population should be a priority for cancer service providers.

## Figures and Tables

**Table 1:** Model inputs, mean, and sensitivity values and sources.

Description	Mean	Low	High	Source
Age entering the cycle (years)	52	44	60	EfH trial, Hayes 2018 [18]
**Annual transition probabilities**				
Early stage to distant recurrence	0.018	0.016	0.019	Wu 2016 [29]
Early stage to local recurrence	0.007	0.006	0.007	As above
Local recurrence to distant	0.0997	0.0897	0.1097	Wapnir 2006 [30] & Anderson 2009 [31]
Death from all causes (by age)	Table	Values differ by age		Supplementary File, Life tables, female [32]
Death from distant recurrence	0.230	0.207	0.253	SEER data [33]
Death from local recurrence	0.069	0.006	0.007	Witteveen 2014 [34]
**Trial-based probabilities**				
Non-BrC mortality for exercise intvn	0.0006	0.0000	0.0032	EfH trial, Hayes 2018 [18]
Non-BrC mortality for usual care	0.0047	0.0015	0.0110	As above
BrC mortality for exercise intvn	0.0059	0.0028	0.0109	As above
BrC mortality for usual care	0.0096	0.0046	0.0176	As above
BrC recurrence in exercise intvn	0.0072	0.0037	0.0125	As above
BrC recurrence in usual care	0.0076	0.0033	0.0150	As above
**Costs (AU$)**				
Exercise intervention	1344	1209	1478	EfH trial, Gordon 2017 [20]
Local recurrence	8679	7811	9547	Verry 2012 [35]
Distant recurrence	27,677	24,900	30,434	As above
BrC survivors’ follow-up care	Table	Values differ by year post dx		Supplementary File [36]
End-of-life—BrC	25,475	22,928	28,023	Reeve 2017 [37]
End of life—other causes	12,122	10,910	13,334	As above
Out-of-pocket expenses (annual)	2538	797	9079	Deloitte 2016 [38], first 2 years only
**Costs of productivity losses (AU$) from**				
Premature death from breast cancer	149,909	134,918	164,900	Carter 2016 [39]
Distant recurrence	34,719	31,248	38,191	Deloitte 2016 [38,40]
Local recurrence	22,785	20,506	25,063	As above
BrC early stage	10,850	9765	11,935	As above
Carers for metastases	56,419	50,777	62,061	As above
Carers for locoregional cancer	29,295	26,365	32,224	As above
Carers for no recurrence/early stage	2170	1953	2387	As above
**Health utilities (quality of life)**				
Utility for women at baseline	0.818	0.718	0.918	Paracha 2016 [41]
Additional utility with exercise program	0.070	0.040	0.10	EfH trial, Gordon 2017 [20], first year only
Additional utility with usual care	0.020	0.000	0.06	As above
Utility for local recurrence	0.670	0.567	0.767	Paracha 2016 [41]
Utility for distant recurrence	0.640	0.540	0.74	“
Utility for terminal BrC	0.514	0.414	0.614	“

ABS = Australian Bureau of Statistics; BrC = breast cancer; dx = diagnosis; EfH = exercise for health; intvn = intervention; SEER = surveillance, epidemiology, and end results.

**Table 2:** Main results for costs $AU, quality-adjusted life years and life-years.

	Exercise	Usual Care	Incremental	95% UI
	Mean	Mean	Difference	
Costs	$281,445	$274,035	$7409	Cost-saving, $16,275
QALYs	10.97	10.63	0.35	0.20, 0.52
Life-years	25.64	24.82	0.82	0.39, 1.4
Incremental cost per QALY	-	-	$21,247	Dominant ^1^, $31,398
Incremental cost per life-year saved	-	-	$8894	Dominant ^1^, $11,769

Dominant means cost saving and higher health effects. UI = uncertainty interval, QALY = quality-adjusted life year. ^1^ Dominant means the exercise group resulted in cost savings and improved QALYs or life years.

## Supplementary Materials

The following are available online at https://www.mdpi.com/1660-4601/17/22/8608/s1. Table S1. Detail of intervention delivery costs. Table S2: Productivity losses, end-of-life healthcare costs, and out-of-pocket costs. Table S3: Costs for recurrence of breast cancer. Table S4: Follow-up schedule. Table S5: Probability of local and distant recurrence. Table S6: Probability of locoregional disease progressing to distant disease. Table S7: Survival rates from breast cancer by stage. Table S8: Mortality and recurrence rates for exercise and usual care groups (source EfH trial). Table S9: Probability of death by age (female).
