# Supplementary material for: Cost-Effectiveness Analysis from a Randomized Controlled Trial of Tailored Exercise Prescription for Women with Breast Cancer with 8-Year Follow-Up
Source: Int J Environ Res Public Health. 2020 Nov 19;17(22):8608. doi: 10.3390/ijerph17228608 (PMC7699530; doi:10.3390/ijerph17228608)
Supplement: Supplementary file 1 [file ijerph-17-08608-s001.pdf]

# Supplementary File

Title: Cost-Effectiveness Analysis from a Randomized Controlled Trial of Tailored Exercise Prescription for Women with Breast Cancer with 8-Year Follow Up

**Table S1.** Detail of intervention delivery costs.

| Personnel | Details & Sources | Lower | Average | Upper |
|---|---|---|---|---|
| EP visits: AEP: Professional Staff, HEW 5, Level 3, casual (source: QUT) | $45.77/hour, 20 hours/participant: includes exercise sessions 16 each, reporting, follow-up http://www2.qut.edu.au/jobs/working-at-qut/docs/Salary%20Scales%20Professional%20Staff.pdf | | 915.40 | |
| Admin Staff : Professional Staff, HEW 4, Level 1, casual (source: QUT) | $39.57/hour, 3.5 hours/week for 16 weeks = 56 hours for 134 participants / estimated as 0.5–1 hour for per participant | 19.79 | 29.68 | 39.57 |
| EP visits using MBS item(government as the payer) | 10 visits *item 10953*$62.25 + care plan(721+723) *2 | | 1139.60 | |
| **Intervention Materials** | **Details** | Lower | Average | Upper |
| Workbook, Tracker, CMF (pack) | Officework Printing service (2018) 1/participant https://www.officeworks.com.au/resources/pdfs/guides/print-and-copy_price-guide.pdf | | | |
| Educational Workbook | 54 colour pages on double sided 100 gsm, A4 $0.23-$0.69 per side | 12.42 | 14.15 | 15.87 |
| Exercise Tracker | 2 colour pages on double sided 100 gsm, A4$0.23–$0.69 per side | 0.46 | 0.92 | 1.38 |
| Case Management Folder | 33 black and white pages on double sided 100 gsm, A4 *$0.04–$0.10 | 1.32 | 2.31 | 3.3 |
| Total | | **14.20** | 17.38 | **20.55** |
| **Resources and other** | **Details** | | | |
| Equipment/Clothes purchased by patients | $40.46 per patient / 8months * inflation Gordon et al. 2017 | | 42.40 | |
| **Intervention delivery** | **Intervention delivery** | | | |
| Office rental | $10/sq m per week x 15 sq m for 8 months (34.66 weeks) * inflation from 2014 (4.97%) devided by 67 participants Gordon et al. 2017 | | 81.32 | |
| Mobile plan | Telephone calls for follow-up, reminders, etc. $50–$100 per month *8months/50 paticipants | 8.00 | 12.00 | 16.00 |
| | 2018 | low | 1322.38 | high |
| | CPI inflation to 2019 = 1.61% to bring to 2019 dollars with ±10% low and high values | 1209.30 | 1343.67 | 1478.03 |

**Table S2.** Productivity losses, end-of-life healthcare costs, and out-of-pocket costs.

| Items | Note | Costs | | |
|---|---|---|---|---|
| | | Early stage | Locoregional Recurrence | Metastasis |
| **Productivity losses - households / carers** | | | | |
| Household work time loss—Hours worked by Household; hours worked prior to cancer diagnosis—post-treatment2016, Deloitte report ,Financial impacts of breast cancer in Australia | | 2.00 | 27.00 | 52.00 |
| Household productivity loss (per week) Work time loss* average wage Locoregional is average of no recurrence (profile 1) and metastasis (profile 5) | | 79.77 | 1076.87 | 2073.98 |
| | Approx 90.9% of EfH participants were under 65 working age | 72.51 | 978.88 | 1885.25 |
| | 74.5% of women are in the work force | 54.02 | 729.26 | 1404.51 |
| | 54.5% work FT | 29.44 | 397.45 | 765.46 |
| | plus 45.5% work PT (assume 50%) | 41.73 | 563.36 | 1084.98 |
| Households' productivity loss (annual ) | 10% | $2169.97 | $29,294.54 | $56,419.12 |
| | Low 10% | $1952.97 | $26,365.09 | $50,777.21 |
| | High 10% | $2386.96 | $32,224.00 | $62,061.04 |
| Average Salary$1515.60/week ,full-time work load,2016 ABS #6306https://www.abs.gov.au/AUSSTATS/abs@.nsf/Lookup/6306.0Main+Features1May%202018?OpenDocument | **Average hourly rate=weekly rate/38 hour** | **39.88** | | |
| **Productivity losses - Individuals** | | | | |
| Patients' productivity loss (hrs per week) Survivors: hours worked prior to cancer diagnosis - post-treatment; Recurrence: hours worked prior to cancer diagnosis - during treatment 2016, Deloitte report ,Financial impacts of breast cancer in Australia | Survivors: Urban:28−16 = 14hours, Non-urban: 25−3 = 22 hours; Recurrence: Urban:28−0 = 28hours, Non-urban: 25−0 = 25 hours; | 10.00 | 21.00 | 32.00 |
| Patients' productivity loss (annual) Work time loss* average wage Locoregional is average of no recurrence (profile 1) and metastasis (profile 5) | | 398.84 | 837.57 | 1276.29 |
| | Approx 90.9% of EfH participants were under 65 working age | 362.55 | 761.35 | 1160.15 |
| ABS 2020 | 74.5% of women are in the work force | 270.10 | 567.21 | 864.31 |

| Items | Note | Costs | | |
|---|---|---|---|---|
| | 54.5% work FT | 147.20 | 309.13 | 471.05 |
| | plus 45.5% work PT (assume 50%) | 208.65 | 438.17 | 667.68 |
| Subtotal | 10% | $10,849.83 | $22,784.65 | $34,719.46 |
| | Low 10% | $9764.85 | $20,506.18 | $31,247.51 |
| | High 10% | $11,934.81 | $25,063.11 | $38,191.41 |
| | Applies to less than 65 years old | | | |
| **End-of-life healthcare costs** | | Average | Lower | Upper |
| Last six months of life, these costs accrue, based on Australian data linkage study Reeve et al. 2018 (adjusted to 2017 AUD) | Cancer cohort | $25,475.47 | $22,927.92 | $28,023.02 |
| | Comparison cohort - non cancer | $12,122.00 | $10,909.80 | $13,334.20 |
| https://www.rba.gov.au/statistics/historical-data.html#exchange-rates | From 2010-2019RBA inflation calculator | 19.8% | | |
| | | | 10% | |
| **Lost income due to premature death** | | 2019 | Lower | Upper |
| Individual productivity loss Carter 2016 | Applies to less than 65 years old | $149,909 | $134,918.46 | $164,900.34 |
| https://www.rba.gov.au/calculator/annualDecimal.html | RBA inflation calculator | 5.57% | | |
| **Out of pocket costs due to breast cancer** | | | | |
| Costs for two year period where most OOPs accrue, Deloitte Access Economics report 2016, found molecular profile did not sign change costs in stage I-III--2016, Deloitte report ,Financial impacts of breast cancer in Australia | | 2019 | | |
| | 2 years | $5076.86 | $1594.11 | $18,158.04 |
| | Convert to 1 year | $2538.43 | $797.05 | $9079.02 |
| | This cost for 2 years then 25% of cost thereafter | | | |

**Table S3.** Costs for recurrence of breast cancer.

| | Probability | Costs | Costs (2018 AUD) | Total Costs | Sources | |
|---|---|---|---|---|---|---|
| **Local Recurrence** | | | | | | |
| Inpatient major surgical procedure AR-DRG(6393.00) | 100% | 6393 | 7160.16 | 7160.16 | Verry et al. 2012 | Inflated 2011–2018 |
| Radiotherapy (13%)- 13% of local recurrence patients receive radiotherapy | 13% | 5171.75 | 5792.36 | 753.0068 | | |
| Radiotherapy costs include an initial and follow-up consultation, computed tomography planning and megavoltage – three fields (breast, boost and axilla) for 6 weeks | | | | | | |
| Specialist visits *4 - item 104(85.55)*4 | 100% | | 342.2 | 342.2 | | |
| GP visits*4 - item 36(71.7)*4 | 100% | | 286.8 | 286.8 | | |
| **Total** | | | | 8542.1668 | Lower | Upper |
| | | | 2018 to 2019 | $8678.84 | $7810.96 | $9546.73 |
| **Distant metastases (annual)** | | | | | | |
| Inpatient procedure*3 (70%) 70% receive procedure | 70% | 5190.33 | 5813.1696 | 12207.65616 | | |
| Third-generation chemotherapy (100%)(MBS, PBS) | 100% | 5419.06 | 6069.3472 | 6069.3472 | | |
| Fourth-generation chemotherapy (50%)50% receive | 50% | 15172.3 | 16992.9872 | 8496.4936 | | |
| Specialist visits*2- item 104(85.55)*2 | 100% | | 171.1 | 171.1 | | |
| GP visits *4 - item 36(71.7)*4 | 100% | | 286.8 | 286.8 | | |
| **Total** | | | | 27231.39696 | Lower | Upper |
| | | | | $27,667.10 | $24,900.39 | $30,433.81 |

**Table S4.** Follow-up schedule.

| | Frequency | Costs | | |
|---|---|---|---|---|
| | | lower | medium | upper |
| Year 1–2 | | | | |
| Specialist Visits | 3–6 months | 171.1 | 256.65 | 342.2 |
| GP visits | 3–6 months | 143.4 | 215.1 | 286.8 |
| Mammography | annually | 71.6 | $89.50 | 107.4 |
| Total | | 386.1 | 561.25 | 736.4 |
| Year 3–5 | | | | |
| Specialist Visits | 6–12 months | 85.55 | 128.325 | 171.1 |
| GP visits | 6–12 months | 71.7 | 107.55 | 143.4 |

| | | | | |
|---|---|---|---|---|
| Mammography | annually | 71.6 | $89.50 | 107.4 |
| Total | | 228.85 | 325.375 | 421.9 |
| After year 5 | | | | |
| Specialist Visits | annually | 68.44 | 85.55 | 102.66 |
| GP visits | annually | 57.36 | 71.7 | 86.04 |
| Mammography | annually | 71.6 | $89.50 | 107.4 |
| Total | | 197.4 | 246.75 | 296.1 |

**Table S5.** Probability of local and distant recurrence.

| | Prob | Rate | 1-year prob | 10% low | 10% high | Source |
|---|---|---|---|---|---|---|
| Local Recurrence - 6.9year follow-up | 0.044118 | 0.006539194 | 0.0065179 | 0.005866074 | 0.007169645 | Wu et al. 2016; retrospective, 1088 records, 6.9y follow-up, Canada |
| Distant Recurrence 6.9year follow-up | 0.115809 | 0.017837968 | 0.017680 | 0.015911832 | 0.019447794 | |

**Table S6.** Probability of locoregional disease progressing to distant disease.

| | Disease free prob | Prog prob | Rate | 1-year prob | 10% low | 10% high | Sources |
|---|---|---|---|---|---|---|---|
| Positive nodes | 0.514 | 0.486 | 0.133106403 | 0.124628 | 0.112165 | 0.137091 | Wapnir 2006 |
| Negative nodes | 0.669 | 0.331 | 0.080394244 | 0.077248 | 0.069523 | 0.084972 | Anderson 2009 |
| 57% had negative in study | 0.5915 | 0.4085 | 0.105018719 | 0.099692 | 0.089723 | 0.109662 | |

**Table S7.** Survival rates from breast cancer by stage.

| | Survival Prob | Mortality Prob | Rate | 1-year Prob | 10% low | 10% high | Sources |
|---|---|---|---|---|---|---|---|
| Locoregional Recurrence—10 year | 49.0% | 51.000% | 0.071334989 | 0.068850 | 0.061965 | 0.075735 | Witteveen et al. paper; Netherland;2014 |
| Distant Recurrence—5 year | 27.0% | 73.000% | 0.261866664 | 0.230386 | 0.207348 | 0.253425 | https://seer.cancer.gov/statfacts/html/breast.html |

**Table S8.** Mortality and recurrence rates for exercise and usual care groups (source EfH trial).

| RR for exercise program Survival (measured by mortality) | EfH results | 8-year probability | Rate | 1-year probability |
|---|---|---|---|---|
| Overall mortality—usual care | 15/130 | 0.115384615 | 0.014771364 | 0.014663 |
| Overall mortality—exercise | 11/207 | 0.053140097 | 0.006578811 | 0.006557 |
| Non-breast cancer Death—Usual Care | 5/130 | 0.038461538 | 0.004725387 | 0.004714 |

| | | | | |
|---|---|---|---|---|
| | low 95% CI | 0.0126 | 0.001527717 | 0.001527 |
| | high 95% CI | 0.0875 | 0.011032192 | 0.010972 |
| Non-breast cancer Death—Exercise | 1/207 | 0.004830918 | 0.000583449 | 0.000583 |
| | low 95% CI | 0.0001 | 1.20488E-05 | 0.000012 |
| | high 95% CI | 0.0266 | 0.003248215 | 0.003243 |
| Breast cancer Death—Usual Care | 10/130 | 0.076923077 | 0.009643700 | 0.009597 |
| | low 95% CI | 0.0375 | 0.004604965 | 0.004594 |
| | high 95% CI | 0.1369 | 0.017737918 | 0.017582 |
| Breast cancer Death—Exercise | 10/207 | 0.048309179 | 0.005965670 | 0.005948 |
| | low 95% CI | 0.0234 | 0.002852786 | 0.002849 |
| | high 95% CI | 0.087 | 0.010966193 | 0.010906 |
| | Applied in model up to 8 years thereafter usual all cause death | | | |
| | | | 7.96154 | 2.074622255 |
| **Recurrence rates between Exercise and UC groups** | | | | |
| Disease free survival | | 8-year probability | Rate | 1-year probability |
| Recurrence—Exercise group | 12/207 | 0.057971014 | 0.007195089 | 0.007169 |
| | low 95% CI | 0.0303 | 0.003707052 | 0.003700 |
| | high 95% CI | 0.0991 | 0.012573616 | 0.012495 |
| Recurrence—Usual care | 8/130 | 0.061538462 | 0.007652218 | 0.007623 |
| | low 95% CI | 0.0269 | 0.003285353 | 0.003280 |
| | high 95% CI | 0.1177 | 0.015087126 | 0.014974 |

**Table S9. Probability of death by age (female)** https://www.abs.gov.au/AUSSTATS/abs@.nsf/DetailsPage/3302.0.55.0012016-2018?OpenDocument.

| Age | Prob Death | Age | Prob Death | Age | Prob Death | Age | Prob Death |
|---|---|---|---|---|---|---|---|
| 0 | 0.003025414 | 25 | 0.000239971 | 50 | 0.001788399 | 75 | 0.016630166 |
| 1 | 0.000229974 | 26 | 0.000259966 | 51 | 0.001968061 | 76 | 0.018594941 |
| 2 | 0.000119993 | 27 | 0.000269964 | 52 | 0.002127733 | 77 | 0.020937698 |
| 3 | 0.000099995 | 28 | 0.000279961 | 53 | 0.002307334 | 78 | 0.023724053 |
| 4 | 0.000089996 | 29 | 0.000299955 | 54 | 0.002496878 | 79 | 0.02677502 |
| 5 | 0.000079997 | 30 | 0.000329946 | 55 | 0.002686385 | 80 | 0.030369298 |
| 6 | 0.000069998 | 31 | 0.000369932 | 56 | 0.002875857 | 81 | 0.034635955 |
| 7 | 0.000059998 | 32 | 0.00039992 | 57 | 0.0030952 | 82 | 0.039527569 |
| 8 | 0.000059998 | 33 | 0.000429908 | 58 | 0.003374294 | 83 | 0.045063087 |
| 9 | 0.000059998 | 34 | 0.000479885 | 59 | 0.00365331 | 84 | 0.051724304 |
| 10 | 0.000059998 | 35 | 0.000519865 | 60 | 0.003982051 | 85 | 0.059101491 |
| 11 | 0.000069998 | 36 | 0.000549849 | 61 | 0.004310682 | 86 | 0.067298439 |

| | | | | | | | |
|---|---|---|---|---|---|---|---|
| 12 | 0.000079997 | 37 | 0.000589826 | 62 | 0.004619298 | 87 | 0.076902116 |
| 13 | 0.000099995 | 38 | 0.000649789 | 63 | 0.004927818 | 88 | 0.087575558 |
| 14 | 0.000129992 | 39 | 0.000699755 | 64 | 0.005276033 | 89 | 0.099450368 |
| 15 | 0.000149989 | 40 | 0.000759711 | 65 | 0.005743443 | 90 | 0.112511753 |
| 16 | 0.000179984 | 41 | 0.000829656 | 66 | 0.006339818 | 91 | 0.126231664 |
| 17 | 0.00019998 | 42 | 0.000919577 | 67 | 0.007074855 | 92 | 0.140461791 |
| 18 | 0.000209978 | 43 | 0.00102947 | 68 | 0.00789864 | 93 | 0.155237706 |
| 19 | 0.000219976 | 44 | 0.001129362 | 69 | 0.008721743 | 94 | 0.170921139 |
| 20 | 0.000229974 | 45 | 0.001209268 | 70 | 0.009623396 | 95 | 0.181351116 |
| 21 | 0.000229974 | 46 | 0.001279181 | 71 | 0.010662746 | 96 | 0.202162232 |
| 22 | 0.000229974 | 47 | 0.001369062 | 72 | 0.011888763 | 97 | 0.217138905 |
| 23 | 0.000229974 | 48 | 0.001508861 | 73 | 0.013330353 | 98 | 0.236147064 |
| 24 | 0.000239971 | 49 | 0.001638656 | 74 | 0.014878209 | 99 | 0.258907626 |
| | | | | | | 100 | 0.279658591 |
